# Antenatal cervical length measurement as a predictor of successful vaginal birth

**DOI:** 10.1186/s12884-020-02878-z

**Published:** 2020-03-30

**Authors:** Omima T. Taha, Mohamed Elprince, Khaled A. Atwa, Asmaa M. Elgedawy, Amal A. Ahmed, Rasha E. Khamees

**Affiliations:** Department of Obstetrics and Gynecology, Faculty of Medicine, Suez Canal University, Round Road, Ismailia, 41111 Egypt

**Keywords:** Cervical length, Prediction, Vaginal delivery

## Abstract

**Background:**

Antenatal cervical length measurement has paramount importance in the prediction of labor. It was compared to the Bishop Score and incorporated in the modified Bishop score due to its relevance and convenience. It is a more accurate tool that imposes no harm or distress to the patients. The study aimed to evaluate the role of antenatal cervical length measurement in the prediction of a successful vaginal birth and its relation to the duration of labor.

**Methods:**

This was a prospective cohort study, conducted at the emergency ward of obstetrics and gynecology department. We recruited 162 women over 1 year from January 2018 to January 2019. Women eligible for the study had a transvaginal ultrasound for the examination of the cervical length before the onset of labor. The success of vaginal delivery was evaluated.

**Results:**

The mean cervical length (mm) was 43.3 ± 8.0. The majority of the patients labored spontaneously [102 (63.0%)] while the remaining ones required induction of labor due to different causes. One hundred and eight patients (66.7%) had a successful vaginal delivery. The cervical length was significantly shorter among patients who delivered vaginally than those delivered by CS (*P*-value < 0.001). Multiple factors had a significant role in the prediction of the mode of delivery (cervical length, BMI, the onset of labor, parity). Maternal body mass index and labor induction were associated with a prolonged duration of the active phase of labor.

**Conclusion:**

Antenatal cervical length measurement predicted the mode of delivery as well as the gestational age at which delivery ensued. It can be used in patients’ counseling regarding the mode of delivery.

## Background

Vaginal delivery is the most important event occurring in women’s life. It carries many risks of significant concerns to the physicians. Predicting the chances of vaginal delivery is of paramount concern for the pregnant woman and her relatives. Laboring women either go into labor spontaneously or undergo induction of labor. Rates of induction of labor have been rising globally, with rates of 26% annually reported in the United States [1]. This required the development of a predictive method for a successful vaginal birth. The most common subjective method of evaluation of the cervix is the Bishop score. Dr. Edward Bishop developed this scoring system and recommended a score ≥ 9 [2] as an indicator of successful induction, which decreased to a score of 6, according to the American College of Obstetrics and gynecology [3]. Bishop score is a subjective method, while transvaginal cervical length measurement is more reliable than digital examination [4]. Transvaginal ultrasound is a safe, accurate, and available tool in all obstetric units. It has an essential predictive as well as a diagnostic role in patients presenting in preterm birth [5]. A significant number of researches predicted the outcome of induction of labor [6], with few studies reporting the relationship of cervical length to the duration of labor. This study was conducted to evaluate the role of cervical length measurement in the prediction of successful vaginal birth and its relation to the duration of labor.

## Methods

This was a prospective cohort study conducted at the labor and delivery ward of the obstetrics and gynecology department of Suez Canal university hospitals. The study included 162 laboring women who attended regular antenatal care and fulfilled the following inclusion and exclusion criteria. Inclusion criteria included singleton pregnancies with an uneventful antenatal course between 37+ 0 and 39+ 0 weeks of gestation. Exclusion criteria included: a) planned cesarean section delivery, b) women presenting in the active phase of labor, c) history of cervical insufficiency, d) history of previous cervical surgery (cone biopsy, large loop excision of the transformation zone (LLETZ), e) previous preterm births, f) women with severe obstetric and medical conditions, g) fetal growth restriction, and h) fetal abnormalities.

Women eligible for the study were asked to participate in this study after obtaining informed written consent. We illustrated the necessity of transvaginal ultrasound to them. Patients were evaluated for their demographic data, including age, parity, body mass index (BMI), occupation, and education level.

Ultrasound was performed using a Mindray DC- 60 machines with a transvaginal probe V 11-3B. A sagittal view of the cervix with no compression was obtained. The cervical length was measured from the internal to the external os with visualization of the entire cervical canal. Measurements were obtained in the antenatal period from 37 to 39 weeks gestation, with the bladder empty. The same investigator did the cervical length measurement for all cases. Three measurements were obtained for the cervical length, and the shortest one was considered in the analysis.

Women were asked to attend to the emergency ward with the start of painful uterine contractions. Upon admission to the labor ward, patients were evaluated for the following items;

- Gestational age at delivery,
- Whether the patient went into labor spontaneously or needed induction of labor,
- duration of the first stage of labor (latent phase **-**defined with the start of painful uterine contractions that are associated with cervical changes either effacement or dilatation up to 4 cm [7] and its time was recorded from the time of admission- and active phase – defined by the presence of regular painful contractions associated with progressive cervical dilatation from 4 cm [7]),
- Duration of the second stage of labor, and
- The mode of delivery at the end.

Induced labor was conducted according to the National Institute of Health and Clinical Excellence (NICE) guidelines using prostaglandin E2 tablets administered vaginally once every 6 h for a maximum of 2 doses. Failed induction was managed after patient counseling with either a further attempt to induce labor or CS delivery [8].

Failure to progress during labor is defined as a cervical dilatation of less than 2 cm in 4 h for first labors and cervical dilatation of less than 2 cm in 4 h or a slowing in the progress of labor for second or subsequent labors. A delay in the second stage of labor is suspected if there were no changes in fetal head descend or rotation for 2 h in nulliparous women and for 1 h in multiparous women. Each situation was dealt with according to the NICE clinical guideline [7].

The eligible women were asked to attend to the emergency and delivery ward at the onset of regular uterine contractions. The duration of the first stage of labor (latent phase and active phase) and the second stage of labor were recorded.

### Statistical analysis

Data were statistically described in terms of mean and standard deviation, frequencies (number of cases), and percentages when appropriate. *P* values less than 0.05 were considered statistically significant. All statistical calculations were done using computer program SPSS (Statistical Package for the Social Science; SPSS Inc., Chicago, IL, USA) release 22 for Microsoft Windows. Parametric tests were used in variables with a normal distribution. Non-normally distributed data were tested using non- parametric tests. Pearson correlation coefficient was calculated between pairs of parametric quantitative variables, and Spearman was calculated for others. Significance was calculated and considered when the *p*-value was found to be less than 0.05.

For survival analysis, Cox regression modeling was done. Univariate modeling was followed by multivariate modeling for the univariate model’s significant variables. The hazard ratio was calculated for each significant factor in the final model reached. A model was fitted with vaginal delivery is the outcome measure.

## Results

A total of 162 patients [66 (40.7%) nulliparous and 96 (59.3%) multiparous women] were recruited. Some of them had pregnancy-induced disorders as gestational diabetes (1/66 in nulliparous and 5/96 in multiparous women) and gestational hypertension (4/66 in nulliparous and 2/96 in multiparous women) (Table 1).

**Table 1:** Demographic data (162 patients)

	Nulli-parous 66/162 (40.7%)	Multi-parous 96/162 (59.3%)	***p***-value
**Age (years)** **(Mean ± SD)**	25 ± 3.6	28.8 ± 4.1	***< 0.001***
**BMI (kg/m**^**2**^**)** **(Mean ± SD)**	27.5 ± 2.3	29 ± 3.4	***0.04***
**Educational status (N %)**			
**None**	0 (0%)	6 (6.2%)	***0.01***
**Middle**	12 (18.2%)	30 (31.3%)	
**High**	54 (81.8%)	60 (62.5%)	
**Cervical length (mm)**			
**(Mean ± SD)**	43.3 ± 9.6	43.3 ± 6.7	0.50
**Median**	43.0	44.0	0.96

The mean gestational age at which delivery ensued was matched in nulliparous and multiparous women. The majority of the patients labored spontaneously (102 (63.0%) with no significant difference among nulliparous and multiparous women. The remaining ones required induction of labor due to different causes (obstetric cholestasis 3.7%, postdate gestation 14.8%, gestational diabetes 3.7%, oligohydramnios 3.7%, and premature rupture of membranes 3.7%). Successful vaginal delivery was achieved in 108/162 patients (66.7%) [30 (45.5%) nulliparous and 78 (81.25%) multiparous women] with an average duration of the different phases of labor (Table 2).

**Table 2:** Obstetric outcomes of the studied population

	Nulli-parous	Multi-parous	***p***-value
**Gestational age at labor (weeks)** **(mean ± SD)**	39.5 ± 1.3	39.3 ± 0.9	0.48
**Mode of delivery**			
**Caesarean section (N %)**	36 (54.5%)	18 (18.8%)	***< 0.001***
**Normal vaginal delivery (N %)**	30 (45.5%)	78 (81.2%)	
**Onset of labor**			
**Spontaneous (N %)**	36 (54.6%)	66 (68.7%)	0.07
**Induced (N %)**	30 (45.4%)	30 (31.3%)	
**Duration of latent phase (hours)** **(mean ± SD)**	6.4 ± 1.1	3.3 ± 2.5	***< 0.001***
**Duration of active phase (hours)** **(mean ± SD)**	4.8 ± 1.9	3.6 ± 1.7	***< 0.001***
**Duration of second stage (minutes)** **(mean ± SD)**	67.5 ± 40.1	27.5 ± 30	***< 0.001***

There were significant associations between cervical length and both onset of labor and mode of delivery in nulli- and multi-parous women (Chi-squared test *p*-value < 0.001 for all) (Table 3).

**Table 3:** Correlation between the cervical length and the obstetric outcomes (nulliparous and multiparous women)

	Nulli-para		Multi-para	
	ρ	*p*-value	ρ	*p*-value
**Gestational age at delivery (weeks)**	0.30	0.01	0.13	0.20
**Duration of the first stage**				
**Latent phase (hours)**	0.16	0.29	0.13	0.24
**Active phase (hours)**	0.02	0.87	0.07	0.50
**Duration of the second stage (minutes)**	0.04	0.81	0.19	0.09

The cervical length was significantly shorter among multiparous women who delivered vaginally than those delivered by cesarean section (CS) (*P*-value < 0.001) (Table 4).

**Table 4:** Cervical length, and mode of delivery

	Cervical length (mm) Mean ± SD	***P*** value
**Nulli-parous**		
**Caesarean section**	44.8 ± 5.9	0.35
**Normal vaginal delivery**	42 ± 11.8	
**Multi-parous**		
**Caesarean section**	45.2 ± 5.6	***< 0.001***
**Normal vaginal delivery**	35 ± 4.2	

Using logistic regression for the prediction of the mode of delivery, all factors (cervical length, BMI, the onset of labor, and parity) were significant in the univariate model as well as the multivariate one (Table 5).

**Table 5:** Univariate and multivariate logistic regression for predicting mode of delivery

Predictor	Univariate			Multivariate		
	OR	95% CI	***P***- value	OR	95% CI	***P***- value
**Cervical length**	0.91	(0.87–0.96)	< 0.001	0.89	(0.84–0.95)	< 0.001
**BMI**	1.16	(1.04–1.29)	0.007	1.14	(1.14–1.52)	< 0.001
**Onset of spontaneous labor**	3.25	(1.64–6.43)	0.001	4.00	(1.77–9.08)	0.001
**Parity**	0.19	(0.10–0.39)	< 0.001	0.54	(0.37–0.77)	0.001

**Fig. 1:**
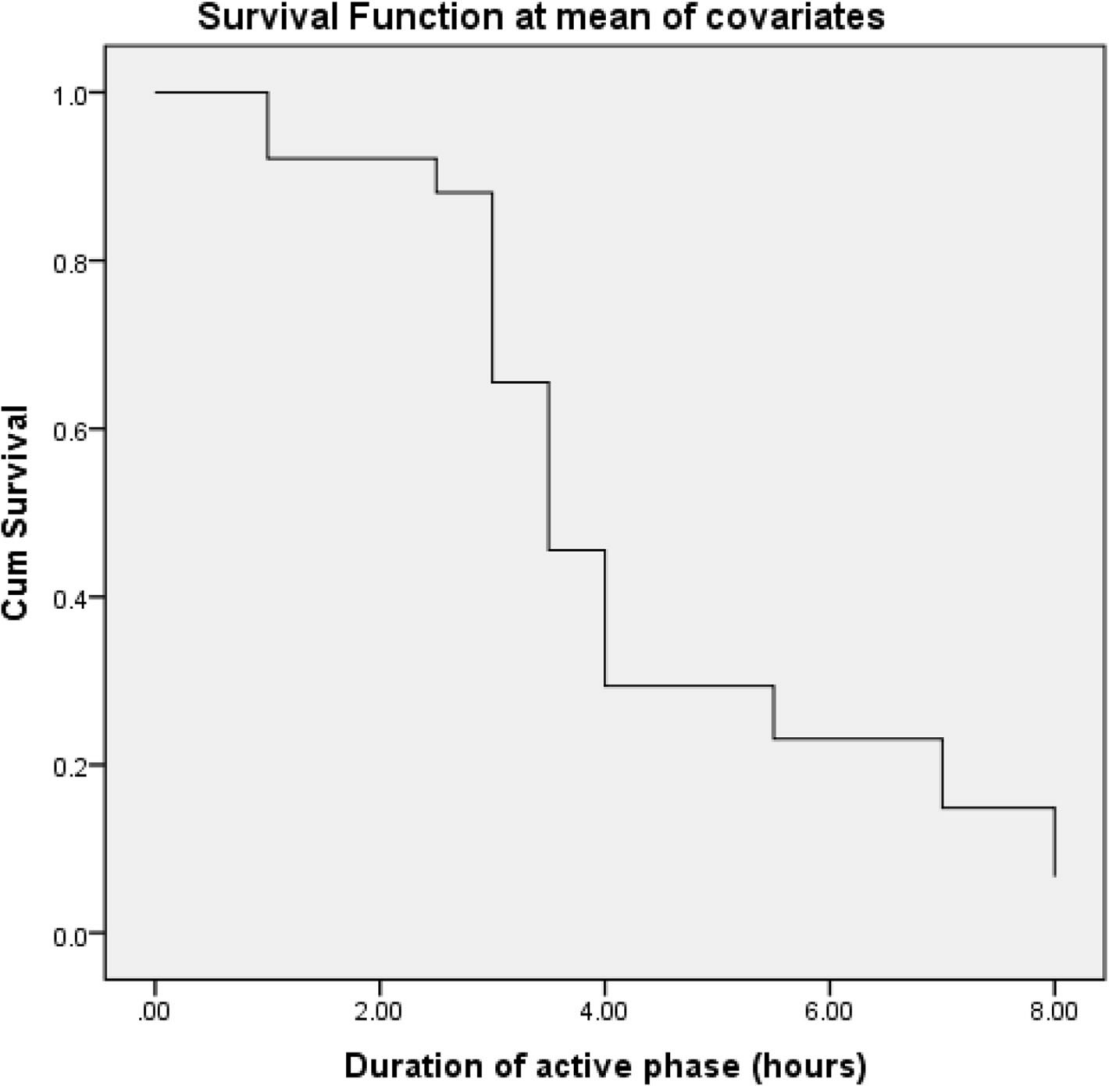
The Cox regression model for women who were to have vaginal delivery

Multiple univariate survival Cox regression models were done to extract the significant factors affecting the duration of the active phase of labor for normal vaginal delivery. A hazard ratio (HR) > 1 illustrates a shorter duration of labor, while an HR < 1 illustrates a longer duration of labor. The Cox regression model for women who were to have vaginal delivery showed that higher BMI and labor induction were lengthening factors (HR 0.845 and 0.580, respectively) and higher parity was a shortening factor (HR 1.353) for the duration of the active phase till vaginal delivery (Fig. 1).

## Discussion

The study revealed that the mean gestational age at the onset of labor was 39.4 ± 1.1 (39.5 ± 1.3 in nulliparous women and 39.3 ± 0.9 in multiparous ones). Also, the cervical length was correlated with the gestational age at delivery (positive correlation) in nulliparous women. This agreed with Donelan et al., who reported prolonged gestation in patients with elongated cervices [9]. Additionally, another study said that cervical length predicted the delay in the onset of labor in women with long cervix significantly; however, they recruited patients in labor pain [10]. They also reported a continued decrease in cervical length as gestation advances. This is explained by the antenatal changes occurring in the cervix and during labor to accomplish complete dilatation, although independent of its length [11].

Induction of labor was required for about one- third of patients (45.45% nulliparous and 31.25% multiparous women), with 14.8% were due to postdate gestation. This was higher than the results reported previously, with 45% of induced labors in their studied population were due to postdate pregnancies [1]. There was a significant association between the cervical length and the onset of labor. The vast majority of patients (66.7%) achieved a successful vaginal birth. CS was required in about one- third of the patients, which was higher than the reported results by El Mekkawi and his colleagues. The shorter cervical length (CL) in most of their studied population could explain this (CL < 28 mm in 143 patients, 122 of them delivered vaginally with a *P*-value of 0.03) [4], which favored successful vaginal birth.

Regarding the mode of delivery, the cervical length differed significantly in those who achieved a vaginal delivery than those who had a CS (*p*-value < 0.001), especially in multiparous women which agreed with previous researches [12, 13]. Also, it was reported in another research that a cervical length (CL) < 28 mm had 87.5% sensitivity, 86.3% specificity, 61.4% positive predictive value, and 96.5% negative predictive value for successful labor induction [4]. Different studies claimed that the cervical length could predict successful labor induction [14, 15]. The cervical length replaces the effacement in the Bishop score, which increases the importance of the cervical length alone or when combined with other factors in the prediction of successful vaginal birth.

In the study performed by Lehner et al., they focused on the correlation between the cervical length and the duration of the first stage of labor. They mentioned that there was no correlation between the cervical length and the duration of labor, which might be reassuring to women with elongated cervices [11]. This was following our findings.

The cervical length was highly predictive of vaginal birth (*P*-value < 0.001), which was also documented by others [1, 16, 17]. This contradicted what was reported by Giyahi et al., who declared that cervical length could not predict the mode of delivery either in univariate or multivariate models [18]. The cervical length cannot be used as a predictor factor for CS alone; it should be combined with other known predictors, as reported by de Vries et al. [12]

A higher BMI and labor induction were lengthening factors (HR 0.845 and 0.580, respectively), and higher parity was a shortening factor (HR 1.353) for the duration of the active phase till vaginal delivery. In a study conducted previously evaluating the effect of maternal weight on the duration of labor in nulliparous women only, the researchers reported that the duration of the active phase of labor was prolonged in overweight women. However, after adjustment for other confounders, the duration of the active labor did not differ significantly [19]. Overall, conflicting results were reported regarding the effect of maternal BMI on the duration of the active phase of labor [20, 21]. Similar results were reported by a previous study, although they recruited women with cervical dilatation of 1 cm, which was considered as the latent phase in this study [22].

### Research implications

The focus on patients undergoing induction of labor would be a source of valuable results, although discussed in previous researches. The evaluation of the cervical length in women undergoing vaginal birth after cesarean (VBAC) needs to be evaluated to provide proper counseling.

### Strengths and limitations of the study

A larger sample size would be more informative. The analyses of subgroups of the studied population (nulliparous and multiparous women) empower the results. The small number of patients who had induction of labor in this study hindered proper analyses.

## Conclusion

Antenatal cervical length measurement was found to predict the mode of delivery as well as the gestational age at which delivery ensued. It can be used in patients’ counseling regarding the mode of delivery. Women would be informed that a successful vaginal birth would be anticipated in women with short cervices; however, the duration of labor would not be affected with elongated cervices.

## Data Availability

The datasets used and/or analyzed during the current study available from the corresponding author on reasonable request. All data generated or analyzed during this study are included in this published article.

## Abbreviations

BMI: Body mass index
CL: Cervical length
CS: Cesarean section
HR: Hazard ratio
LLETZ: Large loop excision of the transformation zone
NICE: National Institute of Health and Clinical Excellence
SPSS: Statistical Package for the Social Science
VBAC: Vaginal birth after cesarean

## References

[1] Panelli DM, Robinson JN, Kaimal AJ, Terry KL, Yang J, Clapp MA, et al. Using Cervical Dilation to Predict Labor Onset: A Tool for Elective Labor Induction Counseling. Am J Perinatol. 2018. 10.1055/s-0039-1677866 ISSN 0735-1631. 10.1055/s-0039-1677866 30695793

[2] Ezebialu IU, Eke AC, Eleje GU, Nwachukwu CE (2015). Methods for assessing pre-induction cervical ripening. Cochrane Database Syst Rev.

[3] Dixon L, Skinner J, Foureur M (2013). Women’s perspectives of the stages and phases of labor. Midwifery.

[4] El Mekkawi SF, Hanafi S, Khalaf-Allah AE, Abdelazim IA, Mohammed EK (2019). Comparison of transvaginal cervical length and modified Bishop’s score as predictors for labor induction in nulliparous women. Asian Pac J Reprod.

[5] Melamed N, Pittini A, Hiersch L, Yogev Y, Korzeniewski SJ, Romero R (2016). Do serial measurements of cervical length improve the prediction of preterm birth in asymptomatic women with twin gestations?. Am J Obstet Gynecol.

[6] Migliorelli F, Rueda C, Angeles MA, Banos N, Posadas DE, Gratacos E, Palacio M (2019). Cervical consistency index and risk of cesarean delivery after induction of labor at term. Ultrasound Obstet Gynecol.

[7] Intrapartum care for healthy women and babies. National institute for health and care excellence**.** Clinical guideline [CG190] Published date: December 2014. 31820894

[8] Inducing labour. National institute for health and care excellence**.** Clinical guideline [CG70] Published date: July 2008.

[9] Donelan EA, Grobman WA, Miller ES (2015). Association of secondtrimester cervical length with prolonged pregnancy. Obstet Gynecol.

[10] Jaisaby A, Phaliwong P, Prommart S, Smanchat B, Bhamarapravatana K, Suwannarurk K (2019). The accuracy of cervical length for prediction of delivery in term pregnancy patients presenting with labor pain. Siriraj Med J.

[11] Léhner G, Reif P, Avian A, Kollmann M, Lakovschek IC, Lang U, Ulrich D. Does third trimester cervical length predict duration of first stage of labor? Wien Klin Wochenschr. 2019. 10.1007/s00508-019-1527-0. 10.1007/s00508-019-1527-0 31312917

[12] de Vries B, Narayan R, McGeechan K, Santiagu S, Vairavan R, Burke M, et al. Is sonographically measured cervical length at 37 weeks of gestation associated with intrapartum cesarean section? A prospective cohort study. Acta Obstet Gynecol Scand. 2018. 10.1111/aogs.13310. 10.1111/aogs.13310 29450884

[13] Gibreil MM, Elboghdady AA, AMS AL-B (2018). Transvaginal ultrasound measurement of cervical length and posterior cervical angle versus bishop scoring in assessment of induction of labour. The Egyptian Journal of H ospital Medicine.

[14] Ware V, Raynor BD (2000). Transvaginal ultrasonographic cervical measurement as a predictor of successful labor induction. Am J Obstet Gynecol.

[15] Daskalakis G, Thomakos N, Hatziioannou L, Mesogitis S, Papantoniou N, Antsaklis A (2006). Sonographic cervical length measurement before labor induction in term nulliparous women. Fetal Diagn Ther.

[16] Beloosesky R, Khatib N, Ganem N, Matanes E, Ginsberg Y, Divon M, Weiner Z (2018). Cervical length measured before delivery and the success rate of vaginal birth after cesarean (VBAC). J Matern-Fetal & Neonatal Med.

[17] Eggebo TM, Økland I, Heien C, Gjessing LK, Romundstad PL, Salvesen KA (2009). Can ultrasound measurements replace digitally assessed elements of the Bishop score?. Acta Obstetricia et Gynecologica.

[18] Giyahi H, Marsosi V, Faghihzadeh S, Kalbasi M, Lamyian M. Sonographic measurement of cervical length and its relation to the onset of spontaneous labor and the mode of delivery. Natl Med J India. 2018;31(2):70–72. 10.4103/0970-258X.253163 30829220

[19] Ellekjaer KL, Bergholt T, Løkkegaard E (2017). Maternal obesity and its effect on labor duration in nulliparous women: a retrospective observational cohort study. BMC Pregnancy Childbirth.

[20] Carlhall S, Kallen K, Blomberg M (2013). Maternal body mass index and duration of labour. Eur J Obstet Gynecol Reprod Biol.

[21] Hilliard AM, Chauhan SP, Zhao Y, Rankins NC (2012). Effect of obesity on length of labour in nulliparous women. Am J Perinatol.

[22] Kominiarek MA, Zhang J, Vanveldhuisn P, Troendle J, Beaver J, Hibbard JU (2011). Contemporary labour patterns: the impact of maternal body mass index. Am J Obstet Gynecol.

