# Correction: Antenatal cervical length measurement as a predictor of successful vaginal birth

**DOI:** 10.1186/s12884-022-05192-y

**Published:** 2022-11-23

**Authors:** Omima T. Taha, Mohamed Elprince, Khaled A. Atwa, Asmaa M. Elgedawy, Amal A. Ahmed, Rasha E. Khamees

**Affiliations:** Department of Obstetrics and Gynecology, Faculty of Medicine, Suez Canal University, Round Road, Ismailia, 41111 Egypt

**Correction: BMC Pregnancy Childbirth 20, 191 (2020)**

**https://doi.org/10.1186/s12884-020-02878-z**

Following publication of the original article [1], the following corrections should be made:

The first paragraph of the results should read:

A total of 162 patients [66 (40.7%) nulliparous and 96 (59.3%) multiparous women] were recruited (Table 1). Some of them had pregnancy-induced disorders as gestational diabetes (1/66 in nulliparous and 5/96 in multiparous women) and gestational hypertension (4/66 in nulliparous and 2/96 in multiparous women).

In Table 1, the mean cervical length measurement for multiparous women was incorrect. The corrected Table 1﻿:

**Table 1:** Demographic data (162 patients)

	Nulli-parous 66/162 (40.7%)	Multi-parous 96/162 (59.3%)	***p***-value
**Age (years)** **(Mean ± SD)**	25 ± 3.6	28.8 ± 4.1	***< 0.001***
**BMI (kg/m**^**2**^**)** **(Mean ± SD)**	27.5 ± 2.3	29 ± 3.4	***0.04***
**Educational status (N %)**			
**None**	0 (0%)	6 (6.2%)	***0.01***
**Middle**	12 (18.2%)	30 (31.3%)	
**High**	54 (81.8%)	60 (62.5%)	
**Cervical length (mm)**			
**(Mean ± SD)**	43.3 ± 9.6	40.2 ± 6.7	0.50
**Median**	43.0	42.0	0.96

The third paragraph of the results should be replaced with the following:

There were significant associations between cervical length and both onset of labor and mode of delivery in nulli- and multi-parous women (Chi-squared test p-value < 0.001 for all).

Table 3 shows that there was a statistically significant weak positive correlation between cervical length and gestational age at delivery in nulli-parous women.
